# On the Dependency of the Electromechanical Response of Rotary MEMS/NEMS on Their Embedded Flexure Hinges’ Geometry

**DOI:** 10.3390/mi14122229

**Published:** 2023-12-12

**Authors:** Alessio Buzzin, Lorenzo Giannini, Gabriele Bocchetta, Andrea Notargiacomo, Ennio Giovine, Andrea Scorza, Rita Asquini, Giampiero de Cesare, Nicola Pio Belfiore

**Affiliations:** 1 Department of Information Engineering, Electronics and Telecommunications, Sapienza University of Rome, Via Eudossiana 18, 00184 Rome, Italy; lorenzo.giannini@uniroma1.it (L.G.); rita.asquini@uniroma1.it (R.A.); giampiero.decesare@uniroma1.it (G.d.C.); 2 Department of Industrial, Electronic and Mechanical Engineering, Roma Tre University, Via Della Vasca Navale 79, 00146 Rome, Italy; gabriele.bocchetta@uniroma3.it (G.B.); andrea.scorza@uniroma3.it (A.S.); nicolapio.belfiore@uniroma3.it (N.P.B.); 3 Institute of Photonics and Nanotechnologies, National Research Council IFN-CNR, Via Del Fosso Del Cavaliere 100, 00133 Rome, Italy; andrea.notargiacomo@cnr.it (A.N.); ennio.giovine@cnr.it (E.G.)

**Keywords:** MEMS, NEMS, comb-drives, electrostatic actuator, flexure hinge, compliant

## Abstract

This paper investigates how the electromechanical response of MEMS/NEMS devices changes when the geometrical characteristics of their embedded flexural hinges are modified. The research is dedicated particularly to MEMS/NEMS devices which are actuated by means of rotary comb-drives. The electromechanical behavior of a chosen rotary device is assessed by studying the rotation of the end effector, the motion of the comb-drive mobile fingers, the actuator’s maximum operating voltage, and the stress sustained by the flexure when the flexure’s shape, length, and width change. The results are compared with the behavior of a standard revolute joint. Outcomes demonstrate that a linear flexible beam cannot perfectly replace the revolute joint as it induces a translation that strongly facilitates the pull-in phenomenon and significantly increases the risk of ruptures of the comb-drives. On the other hand, results show how curved beams provide a motion that better resembles the revolute motion, preserving the structural integrity of the device and avoiding the pull-in phenomenon. Finally, results also show that the end effector motion approaches most precisely the revolute motion when a fine tuning of the beam’s length and width is performed.

## 1. Introduction

Over the last decades, an increasingly growing demand for highly accurate and finely controlled motion-manipulation devices has been pushed by the fields of bioengineering, medicine, and precision measurement with the aim of targeting increasingly smaller objects [1,2,3,4]. Many researchers are focusing on the miniaturization and optimization of devices such as tweezers and micro-positioners [5,6], aiming at manipulating a new class of irregularly shaped micro- and nano-sized objects, such as bacteria and biological cells [7,8]. The applications of such developments span from minimally invasive surgery to drug delivery and tissue engineering, just to name but a few [9,10].

As an inevitable implication of this downscaling trend [11], the conceptualization of a new device must face many challenges, such as adapting the design to significant changes in its physics, studying new classes of materials specifically suitable for the individual application, and focusing on power consumption and energy efficiency [12,13].

Automated micro-manipulation and finely controlled positioning are possible using microelectromechanical systems (MEMS) and nanoelectromechanical systems (NEMS) thanks to the implementation of manufacturing technologies derived from microelectronics [14,15,16]. Although, at the macro scale, the range of mechanical operations can be widened by increasing the complexity of the mechanism, with relatively higher costs but still using well-established technology for fabrication, conventional kinematic pairs cannot be used in micro and nano devices. In fact, serious fabrication issues arise, and, moreover, phenomena such as mechanical backlash and wear, manufacturing inaccuracies, and friction become increasingly more crucial, leading to the failure of the mechanism. On the other hand, precise motion at small scales cannot be achieved by kinematic pairs as they are not technologically compatible with micro- and nano-scaled manufacturing methods [17]. These problems can be solved by designing an equivalent, compliant, and monolithic mechanism [18]. Compliant mechanisms are made by a single block of material, with flexible parts acting as joints and allowing rigid parts to move, offering the best valid alternative to standard rotary hinges [19]. From a manufacturing point of view, this configuration is perfectly suited for microelectronic technologies and enables the miniaturization of such devices to extreme extents, from macro to micro scale, even to the nano scale [20,21]. Thanks to this concept, structures like compliant micro-hinges [21,22], micromechanisms [23,24], microgrippers [25,26,27,28,29,30], and microrobots [31,32,33,34] have been developed.

The early stages of the design of a compliant mechanism are devoted to the study of the desired mechanism as it is a classic multibody system with kinematic pairs. Taking this as the target pseudo-rigid body mechanism, a corresponding compliant mechanism can be generated, although not uniquely [35], by replacing the kinematic pairs with flexible hinges (i.e., the joint replacement method) [36,37]. In order to obtain a selectively flexible device that behaves as the desired one, the design phase must involve a deep study of the structure’s material and a meticulous refinement of the geometries [38]. As a matter of fact, the device’s behavior, motion accuracy, and precision strongly depend on the kind of flexure hinges and their geometry [39,40].

Given that, typically, application-specific optimization approaches can hardly be generalized, and knowing that every flexure hinge implementation presents peculiar advantages and disadvantages, some of the joint replacement requirements depend on the scale of the mechanism. At the macro scale, classical revolute pairs perform an ideal relative rotation, and a flexible replacement can reasonably be considered good enough to mimic a rotary joint [41,42]. On a micro-scale level, flexure hinges might fail in replicating an ideal relative rotation, yet they lead to neglectable deviations in displacement [43,44]. At smaller scales, replacing the joint can give rise to critical issues. For example, if the device is an electrostatic actuator, an advantage of the extreme downscaling consists of the significant increase in electrostatic force obtained per unit voltage [45,46]. On the other hand, micro-deviations from the ideal displacement can cause ruptures and the failure of a system which has sub-micrometric geometrical clearances and tolerances. In such instances, there is a strong requirement for a thorough refinement of the hinges to achieve accurate motion.

Up to now, not much work has been carried out to study and refine the geometry of flexure hinges to accurately mimic an ideal rotary joint in applications involving rotary comb-drives. For example, placing a linear beam as a flexible joint [47,48,49,50,51] is largely different to placing a curved beam [52], and it produces significant changes in the motion of the rigid parts; other crucial parameters involved in the design are the length and width of the flexure beam and its position on the motion plane [53].

This work concerns a pioneering investigation on the electromechanical response of NEMS rotary mechanisms when the joint replacement method is applied. A nano-scaled electrostatic actuator is selected as the device under test since electrostatic actuators are among the most versatile performing and well-established actuation solutions in the MEMS-NEMS field [54]. The device’s physics and operational features are studied for different design variables, such as the beam’s geometry, length, and width. The bending of each flexure is numerically analyzed to assess its capability to withstand mechanical stresses and to provide the correct motion. The electromechanical behavior of each design variant is compared to the ideal behavior of the revolute joint. The simulated rotation of the end effector is compared to the ideal rotation performed by the standard version.

## 2. Structure and Approach

**Figure 1:**
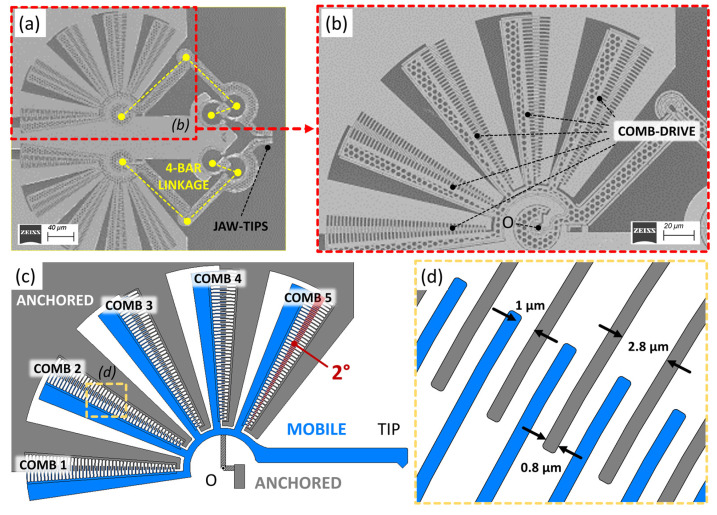
Structure of the device under development: (**a**) scanning electron micrograph of the nano-gripper system, (**b**) enlargement on the actuation part, composed of five comb-drives that provide rotation around the center “O”. (**c**) Top view of the system; voltage is applied between the anchored (gray) and the mobile (blue) fingers of the comb-drives to provide motion of the tip through the hinge, around the center of rotation “O”. (**d**) Detail of the comb-drive fingers.

The structure under test is selected by isolating the actuation part from a more complex device that is currently under fabrication, as shown in the SEM image of Figure 1a. Its operational characteristics have been already extensively demonstrated [55], while Conjugate Surfaces Flexure Hinges (CSFHs) [36] and rotary comb-drives [12,20] have been also widely discussed in the recent past.

The design specifications lead to the selection of monocrystalline silicon as the structural material due to its exceptional mechanical and electrical properties. Mono-crystalline silicon exhibits enhanced elasticity and reduced fragility at the micro scale compared to its macroscopic behavior, making it an ideal choice for MEMS/NEMS applications. Furthermore, monocrystalline silicon offers high mechanical stiffness, which is crucial for the proper functioning of flexure hinges. This stiffness ensures that the hinges can withstand the applied forces and maintain their desired shape, enabling precise and predictable mechanical motion. To further enhance the stiffness and electrical properties of the flexure hinges, a Silicon-on-Insulator (SOI) wafer is selected as the substrate. The SOI wafer provides a high-quality single-crystal silicon layer on a thin insulating layer, which minimizes stress and improves device performances [56]. Moreover, the stiffness of flexure hinges can be theoretically estimated using the compliance matrix approach, as described in [57]. This method allows for the calculation of hinge stiffness based on its geometric parameters and material properties, providing valuable insights into hinge design and optimization.

The fabrication phase requires the implementation of MEMS/NEMS-technology-based techniques. Geometry is transferred on an SOI substrate by lithography; device patterning is performed by reactive ion etching; and, finally, device releasing is achieved by wet etching [58]. In this case, since the smallest feature size is 600 nm and even a minor imperfection could cause unplanned behaviors, electron-beam lithography (EBL) is employed for the geometry definition [59,60,61]. The SEM pictures show the device after the patterning of a hard mask made of chromium, achieved through EBL and the lift-off procedure.

The device under development (Figure 1a) is a nano-gripping system consisting of a double four-bar linkage in a mirrored configuration. Two jaw tips are designed to open and close following a linear trajectory when actuated through two electrostatic rotary actuators. The overall footprint of the device is about (500 × 500) µm^2^. Such complex motion at this scale can be obtained only by means of a fine adjustment of the geometries of the eight hinges. More specifically, by securing the actuators’ motion as close as possible to an ideal rotation, electrostatic failures, ruptures, and malfunctions can be avoided.

Figure 1b shows an enlargement of one of the device’s actuators. Figure 1c depicts the design of the actuator. It is made of a series of rotary comb-drives which provide the in-plane rotation of the actuator’s tip around the center “O”, with the rotation axis perpendicular to the plane. It presents a total footprint of (380 × 230) µm^2^. It consists of five pairs of rotary combs, each pair made of interdigitated fingers. Anchored fingers are fixed to the chip’s substrate (“ANCHORED”, gray in Figure 1c,d), while suspended fingers (“MOBILE”, blue in Figure 1c,d) can move toward the fixed ones when experiencing electrostatic forces due to an applied voltage. Every finger is 0.8 µm wide; the space between the movable and fixed fingers of each pair (i.e., the gap of each capacitor) is 1 µm, as shown in Figure 1d. The two comb-drive teeth arrays have an initial overlap of 2° in neutral state (i.e., 0 V applied). The device thickness is 10 µm.

## 3. Numerical Investigation

When classic revolute joints are replaced by compliant elements, the device mechanical behavior radically changes because elasticity is introduced in the system. More specifically, parasitic motion always appears during hinge inflection, and, for example, an unwanted parasitic translation may affect the desired rotation between the two adjacent parts of the replaced kinematic pair. In case the replaced kinematic pair is actuated by a comb-drive, the parasitic translation gives rise to radial displacements that will cause failure, braking, and radial pull-in phenomena [62]. All these elements must be considered in the design phase in order to avoid very expensive experimental trials.

The literature presents standard analytical models describing deformation, stress, and electrostatic torque as a function of the voltage applied to electrostatic comb-drives. For example, one of the most commonly used models to predict the electrostatic torque in a rotary comb-drive is the following:

(1) $$\tau =\frac{1}{2}\left(\frac{\partial C}{\partial \vartheta }\right)V^{2}=\frac{1}{2}\varepsilon_{0}h\left[\sum_{i=1}^{n-1}{\left(\ln\frac{r_{0}+2i\left(d+g\right)}{r_{0}+2i\left(d+g\right)-g}\right)}^{-1}+\sum_{i=0}^{n-1}{\left(\ln\frac{r_{0}+\left(2i+1\right)\left(d+g\right)}{r_{0}+2i\left(d+g\right)+d}\right)}^{-1}\right]V^{2}$$

This was implemented in [26,49,51], and *τ* represents the electrostatic torque expressed as a function of the voltage *V* applied to the rotary comb-drive and the variation of the equivalent capacitance *C* on the rotation *ϑ*. Here, the variation of *C* depends on the geometric characteristics of the comb-drive, such as the number of fingers *n*, the radius of the first finger *r_0_*, the fingers’ width d, and the fingers’ gap *g*; *ε_0_* represents the vacuum permittivity; h is the thickness of the device. However, this model does not take into account the effect of rotary electrostatic comb-drives on the behavior of uniquely shaped flexure hinges at the investigated scale. For this reason, the proposed analysis relies on simulations performed with COMSOL Multiphysics to better represent this complex interaction with the aim of providing a more complete understanding of the behavior of this class of devices. The “Solid Mechanics” physics module is used, combined with the “Electrostatics” module, to simulate the actual operation of the device and to model the electrostatic force generated by an applied voltage between the anchored and mobile fingers of the comb-drives. Data obtained from different hinge structures are compared to assess their different responses to the applied voltage, their displacement components, and their ability to move the tip of the actuator. Linear and curved flexible beams are modeled and tested. Furthermore, the length and width of the beam are varied to inquire about how such changes affect the tip motion, the mechanical integrity of the system, the maximum operating voltage, and the pull-in phenomenon.

### 3.1. Flexure Hinge: Linear Beam vs. Curved Beam

**Figure 2:**
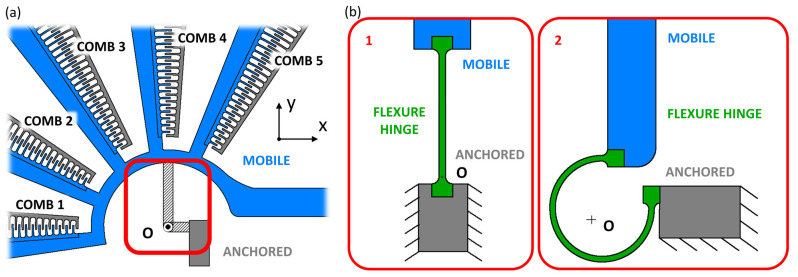
Schematic representation of flexure hinges: (**a**) standard revolute joint in the selected micro-actuator structure replaced by (**b-1**) linear and (**b-2**) curved beam designs.

Figure 2 shows a standard revolute joint (red inset in Figure 2a) connecting the fixed part (“ANCHORED” in the figure) to the suspended part (“MOBILE” in the figure) of a rotary comb-drive; here, the joint is replaced by a linear (Figure 2b-1) and curved (Figure 2b-2) flexure hinge. Both flexure hinges consist of 25 μm long, 10 μm thick, and 0.6 μm wide beams; their deflections are originally meant to induce a rotation of the mobile part around the rotation center “O”. When a linear beam replaces the revolute joint, point O is positioned in correspondence with the anchored section [38], while, in cases where the curved beam is used for the joint replacement, point O will be placed in correspondence with the center of the elastic weights [52,63,64] of a beam, whose axis extends around $\frac{3}{2}\;\pi$ rad (see Figure 2b-2).

It is worth noting that, as Figure 2 shows, the examined flexure hinges have both ends attached to the rigid parts through round-filleted contours with a radius equal to the width of the beam. For this specific application, at this specific scale, other kinds of attachments, such as corner-filleted attachments, may be critical from a technological standpoint; the lithography procedure and the DRIE geometry patterning may cause slight inaccuracies in the corners [65]. Moreover, rounded edges further reduce the risk of mechanical ruptures with respect to sharp edges [66]. For this reason, the fingers of the comb-drives also present round-filleted attachments as they are another critical part of the examined system and increasingly prone to ruptures when miniaturized at such a level. Other types of attachments are not yet considered [67], as this work focuses on shape, length, and width as the main design parameters.

**Figure 3:**
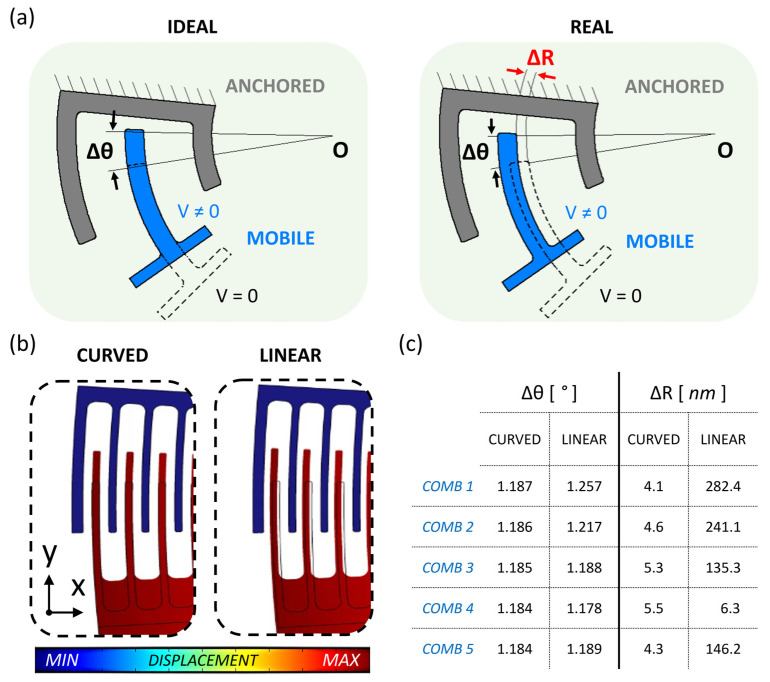
Graphic representation of rotation (Δθ) and radial displacement (ΔR) of the movable comb (mobile) with respect to the fixed comb (anchored) in a rotary comb-drive: (**a**) ideal rotary joint (**left**) compared with flexible joint (**right**) and (**b**) comparison of simulated comb-drive displacement with curved and linear beam as flexure hinge. (**c**) Rotation and radial displacement values of the most external mobile finger of each comb-drive for curved and linear flexure hinges.

The comb-drive fingers’ displacement is monitored, considering “O” as the rotation center in neutral position (0 V applied); the radial displacement of each movable comb during actuation is addressed as Δ*R*, while the rotation of each comb-drive is referred to as Δ*θ*. Figure 3a schematically depicts the displacement of a movable comb-drive’s finger toward the anchored comb when it is subjected to an applied voltage. More specifically, the left part of Figure 3a shows the result of a pure rotation around a classical rotary joint, while the right part of Figure 3a shows the possible outcome of electrostatic actuation through a flexible joint, with Δ*R* added to Δ*θ*. Figure 3b shows the simulated actuator displacement of the curved beam (on the left) and of the linear beam (on the right) as flexure hinges. As expected, the results shown in Figure 3c confirm how both the linear and the curved beams do not provide an exact rotational motion; however, it is also evident that the curved beam is much closer to the target than the linear beam considering the same geometric dimensions. The simulations are carried out at 1.9 V of actuation voltage. Figure 3c reports Δ*θ* and Δ*R* for the most external mobile fingers belonging to each of the five combs, which are numbered clockwise, in both linear flexure hinge and curved flexure hinge cases. The two models are analyzed for an applied voltage of 1.9 V. As a reference, each comb connected to a standard hinge must keep the same Δ*θ* and Δ*R* equal to zero. Although the overall rotation could be considered similar in both cases (and equal to about 1.2°), the curved beam provides a radial displacement of the mobile fingers’ Δ*R* of a few nanometers, while the linear beam causes a Δ*R* of up to hundreds of nanometers. Considering that the actuator’s gap is 1 µm, implementing the linear beam significantly increases the risk of pull in and electrostatic failure even at small actuation voltages. Plus, the linear beam introduces an unwanted parasitic translation of the whole structure (i.e., of the center of rotation O) along the x-axis, added to its rotation. This is demonstrated by observing high values of Δ*R*, except for that of comb 4, which is placed along the y-axis and designed to have the major component of the mobile fingers’ motion parallel to the x-axis (see Figure 2a).

Higher differences of Δ*θ* and Δ*R* values can be observed in the case of the linear beam which correspond to higher non-uniformities in the actuator’s motion when a voltage is applied. Starting from the computation of Δ*R* and Δ*θ*, two parameters are defined, respectively:

(2) $$R_{err}=\Delta R_{max}-\Delta R_{min}$$

where *R_err_* indicates the difference between the maximum (Δ*R_max_*) and minimum (Δ*R_min_*) values of the radial displacement and is defined as the radial non-uniformity factor, while:

(3) $$\theta_{err}=\Delta \theta_{max}-\Delta \theta_{min}$$

where *θ_err_* is defined as the rotational non-uniformity factor and refers to the differences in rotation between the maximum (Δ*θ_max_*) and minimum (Δ*θ_min_*) angular displacement of the five combs of the same actuated structure. As a reference, the ideal behavior is represented by a standard rotary joint with both an *R_err_* and *θ_err_* equal to zero (uniform rotation and no translation). Table 1 reports the results, displaying how both non-uniformity factors of the curved beam model are closer to zero with respect to the linear beam model, leading to a higher affinity of the flexible curved beam’s behavior to the standard hinge. The values of the curved beam’s Δ*θ* differ by thousandths of degrees, while the linear beam’s Δ*θ* values differ by about a tenth of degrees. Moreover, the curved beam’s Δ*R* of the single combs is between 4 nm and 5.4 nm, while the linear beam can produce simultaneously radial displacements of the single combs that span from a few nanometers to hundreds of nanometers. A translation factor is also considered and defined as:

(4) $$T=\frac{\Delta R}{\Delta \theta }$$

The translation factor *T* is useful to understand how much the fingers of each comb-drive move with respect to how much they rotate. Taking as reference the standard rotary hinge, which does not allow any translation (*T* = 0), a smaller *T* corresponds to a more ideal displacement.

Table 2 reports the values of *T* for each of the five comb-drives actuated through linear and curved beam deflection. The results show how the curved beam deflection produces a structure translation of a few nanometers per degree of rotation, while the linear beam induces a translation of hundreds of nanometers per degree of rotation, leading to higher risks of failure. In addition, note that the small value calculated in the case of the comb 4 is a consequence of the values shown in Figure 3c; the whole structure (i.e., the center of rotation O) translates along the x-axis, providing low *T* only if the comb is placed along the y-axis, and its mobile fingers are designed to have the major component of the motion parallel to the x-axis.

**Figure 4:**
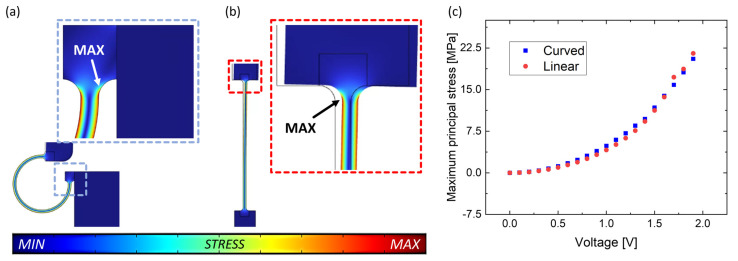
Comparison of simulated Maximum Principal Stress (MPS) for actuation via (**a**) curved beam and (**b**) linear beam and (**c**) trend of MPS values with respect to the applied voltage.

An analysis of the mechanical stresses induced on the flexure hinges by the applied voltage is here reported, taking into account the brittle nature of monocrystalline silicon, as having a Young’s modulus equal to 169 GPa and a yield strength equal to 6.9 GPa before failure [68,69]. The Maximum Principal Stress (MPS) with respect to the applied voltage is reported in Figure 4. When 1.9 V is applied, the MPS sustained by the linear beam is 21.5 MPa, while the MPS sustained by the curved beam is equal to 20.5 MPa. In both cases, the stress is two orders of magnitude lower than the yield stress, and both beams’ rupture is prevented.

**Figure 5:**
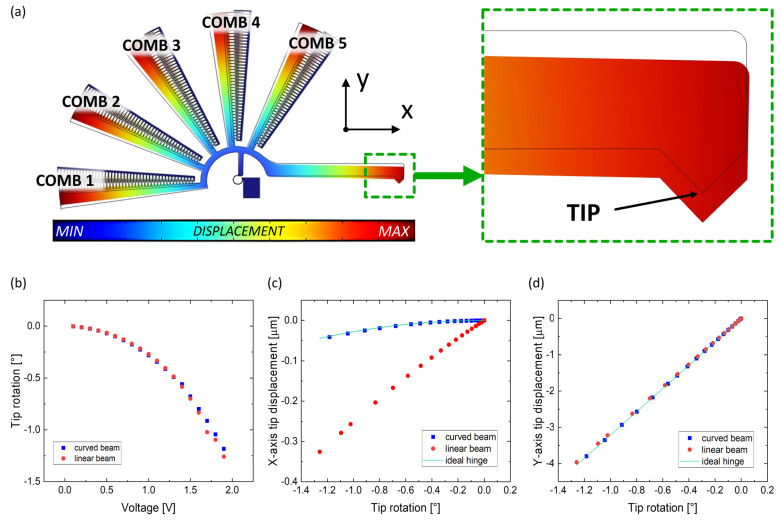
Graphical representation of the displacement made by the device: (**a**) maximum displacement obtained at 1.9 V when the curved beam is implemented as flexure hinge, with an enlargement on the tip, (**b**) tip rotation as a function of the applied voltage and tip displacement along (**c**) x-axis and (**d**) y-axis vs. tip rotation compared to an ideal rotatory joint.

With regards to the analysis of the trajectories assumed by the device for the different actuation voltage values considered during the simulations for both types of flexure hinges, Figure 5a shows the actuator’s displacement when a voltage is applied to the combs and when the curved beam is implemented as a flexure hinge. The framed sketch refers to the structure in neutral position (0 V applied), while the colored sketch refers to the structure’s position when the maximum voltage value considered (1.9 V) is applied. Figure 5b plots the rotation of the actuator’s tip around the center “O” for actuation voltages up to 1.9 V. The plot shows how both linear and curved beams provide the same behavior. Figure 5 also shows the tip displacement along the x-axis (Figure 5c) and y-axis (Figure 5d) with respect to the tip’s rotation for the same voltage sweep previously considered (0 to 1.9 V). Here, the motion provided by the linear (represented by red circles) and curved (blue squares) flexure hinges is compared to the ideal behavior of the same micro-actuator with a standard hinge (green line), revealing how the tip’s movement deviates from the expected behavior. Both flexure hinges perform the same y-axis displacement for an equal degree of rotation while increasing the applied actuation voltage, also nearly equal to the ideal behavior (Figure 5d). However, the x-axis evolution changes significantly (Figure 5c); the curved flexure hinge better resembles a classical hinge, with less displacement along the x-axis for an equal degree of rotation with respect to the linear beam. The results highlight and confirm how the linear flexure hinge differs significantly from the standard hinge, not allowing a pure rotation of the tip but adding a translation component along the x-axis, as also stated when calculating the combs’ Δ*R* and *T* in Figure 3c and Table 3, respectively.

The Maximum Operating Voltage (MOV) of the linear beam model, defined as the maximum value of actuation voltage applicable before reaching the pull-in state and failure of the system, is equal to 1.9 V, while, in the case of the curved beam model, the MOV reaches 2.2 V. Considering that the y-axis displacement of the end effector is similar in the two cases, the curved beam displays a better electromechanical behavior, allowing for more actuation voltage and, therefore, more displacement.

### 3.2. Changing the Beam’s Length

**Figure 6:**
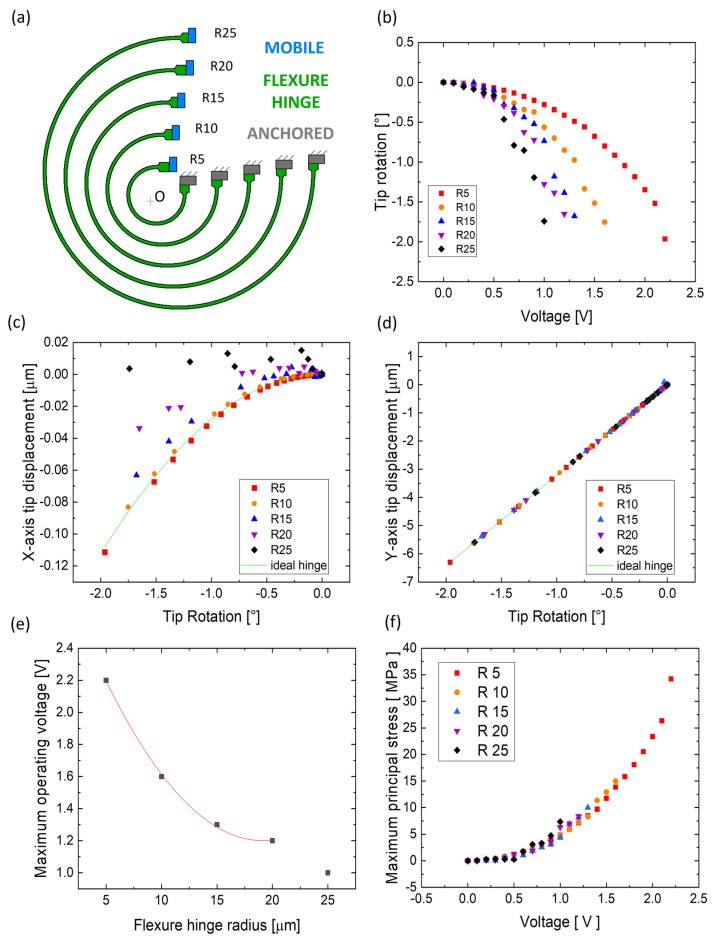
Comparison of the five curved beams with different lengths: (**a**) schematical comparison considering all hinges shaped as three-quarters of a circle’s arc with radii equal to 5, 10, 15, 20, and 25 μm, corresponding to beam lengths of 25, 48.5, 72, 96, and 119 μm, respectively; (**b**) tip rotation as a function of the applied voltage and tip displacement along (**c**) x-axis and (**d**) y-axis vs. tip rotation compared to an ideal rotatory joint; (**e**) maximum operating voltage of the actuator before reaching the pull-in state with respect to the flexure hinge’s radius; (**f**) MPS as a function of the applied voltage.

Along with its geometry, the behavior of a flexure hinge depends on various parameters, one of them being the length of the flexible beam [47]. Figure 6a depicts a flexure hinge with five different design variants with the same geometrical shape (curved beam, three-quarters of a circle), thickness (10 µm), and width (0.6 μm) and radii equal to 5 μm, 10 μm, 15 μm, 20 μm, and 25 μm, corresponding to a neutral axis length of the beams of 25 μm (“R5”), 48.5 μm (“R10”), 72 μm (“R15”), 96 μm (“R20”), and 119 μm (“R25”), respectively. The center of the elastic weights of each curved beam is considered as the center of rotation (“O”) of the device.

Figure 6b plots the actuator’s tip rotation around the “O” with respect to the applied voltage when implementing the five different beam designs; as the beam’s length increases, the same amount of rotation can be achieved with less electrostatic force applied to the combs, i.e., lower voltage. Furthermore, though each of the five behaviors can be considered quadratic, increasingly longer beams display increasingly less smooth trends but allow for greater rotation. As an example, the shortest (25 μm long) beam among those considered allows for a 0.25° tip rotation when 1 V is applied to the combs, while 1.75° tip rotation is achieved when the same voltage is applied to the structure implementing the longest (119 μm long) beam.

Figure 6c,d reports the simulated displacement of the tip along the x- and y-axis per degree of rotation for each of the five variants when the structure is actuated. In the case of y-axis displacement, the observed behavior is increasingly closer to the ideal rotary hinge (green line) for increasingly shorter beams. On the contrary, increasingly longer beams produce bigger discrepancies along the x-axis (i.e., a greater translation component added to the rotation). However, it is worth noting that the difference between x-axis displacement and the ideal case is in the order of a few nanometers per degree of rotation for all five designs, a significantly better scenario when compared to the hundreds of nanometers observed for the linear flexure hinge (Figure 5c).

Figure 6e plots the MOV of the actuator before reaching the pull-in state for all the different beam designs. The trend is quadratic, and the MOV decreases as the beam’s length increases, reaching 1.2 V when the 96 μm long beam (“R20”) is implemented and reaching 1 V when the 119 μm long beam (“R25”) is implemented; nevertheless, the odd displacements and oscillations (Figure 6c) found for the R25 configuration make its results unreliable, excluding it from the curve fitting in Figure 6e.

Figure 6f plots the MPS with respect to the applied voltage for the five different cases. Considering that each evaluation ends at its specific MOV, the whole operational voltage window before the combs’ failure is characterized by an MPS of tens of MPa in the worst-case scenario, well below the silicon’s yield strength. Moreover, each case’s maximum displacement occurs at each case’s MOV, and corresponds to the maximum rotation range, which can reach up to 2° in the “R5” case, more than the 1.25° achievable in the linear case (Figure 5b–d).

Although one of the crucial factors in securing the quality of a simulation campaign is the mesh pattern, which, in this case, is refined down to 50 nm subdomains in the critical parts (flexure hinge, comb-drive fingers, and spacing between the fingers), this anomaly can be ascribed to computing issues and will be addressed in the future by further optimizing the mesh-generation phase.

**Figure 7:**
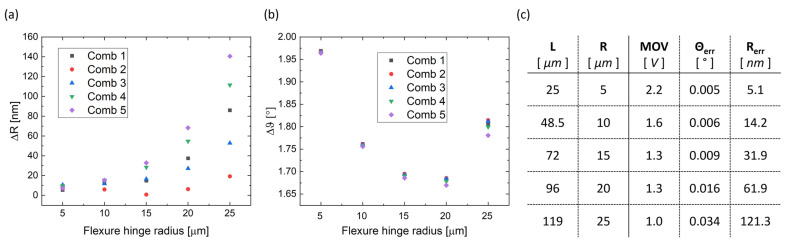
Graphical representation of the results obtained for different lengths of the curved beam: (**a**) angular displacement Δ*θ* of each comb-drive for the five different designs with a radius of 5 μm, 10 μm, 15 μm, 20 μm, and 25 μm and (**b**) radial displacement Δ*R* of each comb-drive for the five different designs. (**c**) Values of *θ_err_* and *R_err_* produced by each design at their MOV.

As in the previous subsection, the displacement of the combs’ fingers is here reported when the curved beam’s length changes. Figure 7 shows Δ*R* and Δ*θ* covered by the single combs for each of the five beam designs, calculated at each version’s MOV. The values of Δ*θ* (Figure 7a) are close to each other, while the values of Δ*R* (Figure 7b) display an increasing span as the radius increases. As shown in Figure 7c, shorter beams exhibit a rotational non-uniformity factor *θ_err_* of thousandths of degrees and a radial non-uniformity factor *θ_err_* of a few nanometers, while longer beams have a *θ_err_* of hundredths of degrees and reach up to hundreds of nanometers of *R_err_*.

The translation factor *T* of each comb-drive is calculated in the five cases (obtained considering the actuator’s MOV for each case). Table 3 shows how longer beams provide higher radial displacement per unit degree of rotation. However, in this case, the structures can reach up to tens of nm/° of *T* when the curved beam is 119 µm long, and, despite its instability, it is still a significantly better scenario when compared to the 25 µm long linear beam that causes values of hundreds of nm/° for *T* (Table 2). In addition, relatively low values can be observed in the case of comb 2 with respect to the other combs, suggesting that the parasitic translation of the whole structure (i.e., the translation of the center of rotation O) takes place in a direction which is close to the direction of the major component of the motion of the comb 2 mobile fingers.

### 3.3. Changing the Beam’s Width

**Figure 8:**
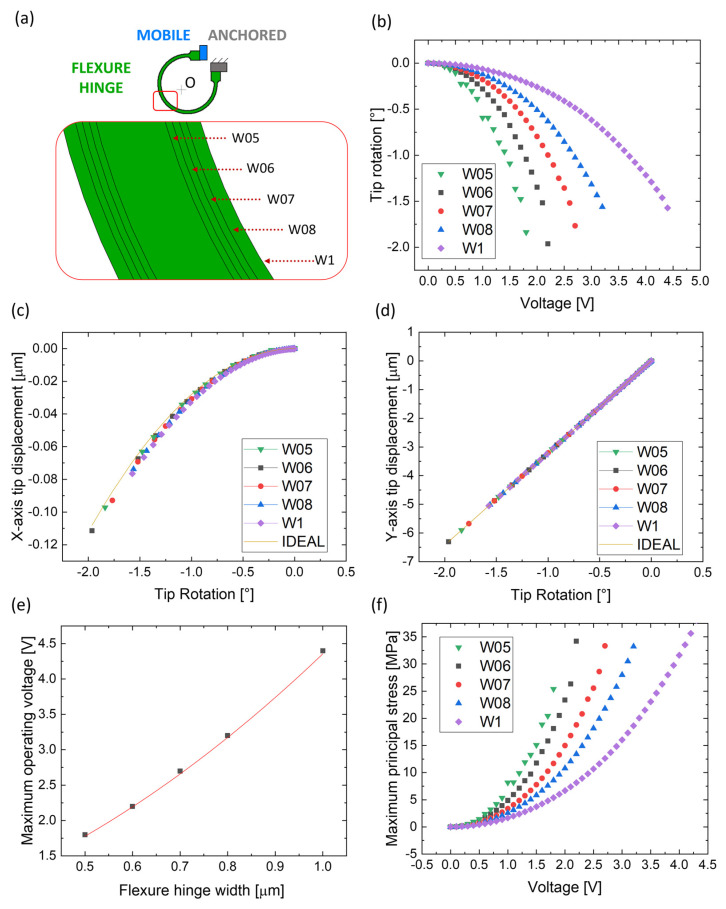
Comparison of the five curved beams with different widths: (**a**) schematical comparison considering all hinges shaped as three-quarters of a circle’s arc with a width of 0.5 μm (“W05”), 0.6 μm (“W06”), 0.7 μm (“W07”), 0.8 μm (“W08”), and 1 μm (“W1”) and a fixed length of 25 μm; (**b**) tip rotation as a function of the applied voltage and tip displacement along (**c**) x-axis and (**d**) y-axis vs. tip rotation compared to an ideal rotatory joint; (**e**) maximum operating voltage of the actuator before reaching the pull-in state with respect to the flexure hinge’s radius; (**f**) MPS as a function of the applied voltage.

Another important parameter to focus on when designing a flexure hinge is the flexible beam’s width. Here, a curved beam is shaped as three-quarters of a circle arc, as in the previous sections; the length of the neutral axis is fixed as 25 μm (corresponding to a radius of 5 μm), and the thickness is equal to 10 μm, while the width W is changed to 0.5 μm, 0.6 μm, 0.7 μm, 0.8 μm, and 1 μm, as schematically depicted in Figure 8a.

Similarly to the previous section, the tip’s motion is here analyzed when implementing the five beam variants as flexure hinges. Figure 8b plots the actuator’s tip rotation around “O” with respect to the applied voltage when the flexure hinge is implemented in these five cases.

The plot confirms how a structure actuated through wider beams requires more energy for its bending; the 0.5 μm wide beam allows for a 1.5° tip rotation with less than 1.5 V applied to the combs, while 3.5 V is needed for the same rotation through the bending of the 1 μm wide beam. Figure 8c,d reports the displacement of the tip along the x- and y-axis per degree of rotation in the five cases. Here, the five structures deviate from the ideal rotary hinge (yellow line) in a less significant manner with respect to the previous analysis, especially along the x-axis (Figure 8c).

Figure 8e plots the MOV of the actuator before reaching the pull-in state in the five cases. The trend is quadratic, and the maximum voltage value increases as the beam’s width increases, spanning from 1.75 V when the 0.5 μm wide beam (“W05”) is implemented to 4.5 V when the 1 μm wide beam (“W1”) is implemented. Figure 8f plots the MPS with respect to the applied voltage. Considering that each evaluation ends at its specific MOV, the whole operational voltage window before failure is characterized by an MPS of tens of MPa, well below the silicon’s yield strength. Finally, as discussed above, the maximum displacement (occurring at each case’s MOV) corresponds to the maximum rotation range, which here can reach up to 2° in the “W06” case.

**Figure 9:**
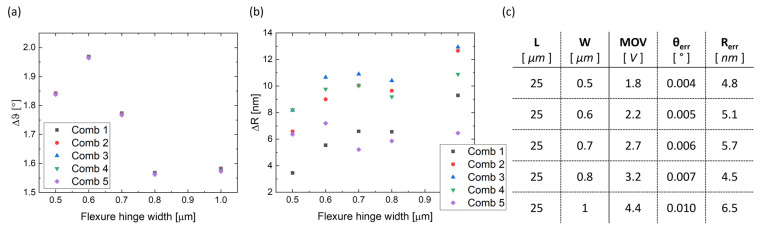
Graphical representation of the results obtained for different widths of the curved beam: (**a**) angular displacement Δθ of each comb-drive for the five different designs with a width of 0.5 μm, 0.6 μm, 0.7 μm, 0.8 μm, and 1 μm and (**b**) radial displacement ΔR of each comb-drive for the five different designs. (**c**) Values of *θ*_*err*_ and *R*_*err*_ produced by each design at their MOV.

As for Figure 7, Figure 9 reports Δ*R*, Δ*θ*, *θ_err_* and *R_err_* for each design, calculated at each MOV.

As in the previous subsection, the translation factor T of each comb-drive in the five different cases is derived and is reported in Table 4. The calculated T is in the order of a few nanometers per degree of rotation for each comb in each of the five different cases. The table shows that T slightly increases when implementing thicker beams as hinges.

## 4. Discussion

Although the replacement of kinematic pairs with flexible beams (i.e., the joint replacement method) is largely considered to be the most suitable alternative to standard mechanisms for obtaining multiple-hinge MEMS/NEMS devices, to the best of the authors’ knowledge, it seems that limited information is available in the literature as a support for designers to refine the geometry of flexible hinges that are working in cooperation with rotary comb-drives to provide accurate rotation of rigid parts. It seems reasonable to believe that, due to the characteristics and the limits of the early technological process of microfabrication, the simple linear beams nowadays are still the most preferred choice for the purpose of revolute joint replacements. However, this investigation has shown that this choice gives rise to critical issues: first, the motion of a rotary part may largely differ from an ideal rotation; secondly, if the rotation is electrostatically set off by rotary comb-drives, failures and ruptures are highly expected.

## 5. Conclusions

This paper has presented a reasoned comparison of different shapes that can be used as flexure hinge in MEMS/NEMS devices, with specific interest in those revolute hinges that may be used to provide an accurate rotation of rotary comb-drives’ mobile fingers without incurring failures and/or ruptures. Different shapes, lengths, and widths have been tested, while the behavior of the compliant device has been observed and compared to the ideal one.

As a first note, in terms of stress, each version of the studied device maintains its mechanical integrity, as the MPS at the MOV is always kept orders of magnitude below silicon’s yield strength. This assures that the pull-in state occurs before the rupture of the flexure hinge. More specifically, the MPS is primarily dependent on the rectangular cross-section of the flexure hinges. This is evident from Figure 4c, which shows that the curved and linear beams have comparable MPS values, despite their different shapes. Figure 6f also shows that the MPS values are similar for the beams at the same applied voltage because, despite the difference in length, they also share the same cross-section. The MPS decreases with increasing hinge width. Additional discussions about other properties are not present as here the primary goal is assessing the MOV and then securing the mechanical integrity of the device in the whole voltage window. Further studies will include assessments on other figures of merit and features, such as flexure hinges’ maximum load and capability to withstand vibrations.

The paper has provided evidence that curved flexible beams should be adopted instead of linear beams to achieve relative motion that better resembles a rotation. Indeed, basic geometries such as straight beams may cause unwanted translation and uneven, non-uniform motion that may cause a change in the device’s center of rotation depending on the applied voltage. Especially at the investigated scale, these phenomena can easily cause electrostatic failure or, even worse, ruptures of the comb-drive structures. Furthermore, numerical results have shown that curved beams can provide rotational motion with an approximation that is better than the one offered by the linear beam, as long as the center of rotation of the device is placed in correspondence to the center of the elastic weights of the curved beam. When the length of the curved beam is changed but the shape and width are kept the same, a trade-off between maximum tip displacement and more ideal rotation must be considered. Longer beams allow for more rotation but also induce more translation; shorter beams reduce the translation but, in doing so, also limit the rotation. Finally, when the width of the shorter curved beam is changed, the analysis shows relatively smaller differences. In particular, the same actuation voltage causes thinner beams to bend more with respect to thicker beams, resulting in more motion of the end effector. In this case, the adoption of a thinner beam causes an increase in both translation and motion non-uniformities that can be considered negligible, but more issues can arise from a technological point of view. Indeed, a trade-off must be made between the best result obtainable in terms of electromechanical behavior and technological viability. While smaller and thinner beams can be suited for this particular purpose, increasingly smaller features at this scale are more challenging from a manufacturing standpoint. On the other hand, bigger and thicker beams are easier and less expensive to produce but cause less electromechanical efficiency and less uniform motion. Moreover, further studies on the direction of the parasitic translation will be carried out in the future as it has been observed to change when the geometry of the flexure hinge is modified.

## Figures and Tables

**Table 1:** Comparison of non-uniform rotation factor *θ_err_* and radial displacement factor *R_err_* of the actuator for linear and curved beam hinges.

	Linear Beam	Curved Beam
*θ_err_* (°)	0.079	0.003
*R_err_* (nm)	234.8	1.4

**Table 2:** Values of translation factor *T* on each comb for linear and curved beam flexure hinges.

	Translation Factor *T* (nm/°)	
	Linear Beam	Curved Beam
Comb 1	116.3	3.4
Comb 2	198.1	3.9
Comb 3	113.9	4.5
Comb 4	5.3	4.6
Comb 5	122.9	3.6

**Table 3:** Values of translation factor *T* of each comb-drive actuated through flexure hinges of different length at the actuator’s MOV.

R (µm)	MOV (V)	Translation Factor *T* (nm/°)				
		Comb 1	Comb 2	Comb 3	Comb 4	Comb 5
5	2.2	2.8	4.6	5.4	4.9	3.7
10	1.6	7.2	3.4	6.8	8.8	8.7
15	1.3	8.8	0.5	9.8	16.8	19.5
20	1.2	22.3	3.7	16.2	32.6	40.9
25	1.0	47.6	10.6	29.2	61.9	78.9

**Table 4:** Values of translation factor T of each comb-drive actuated through flexure hinges at the actuator’s MOV as the width changes.

W (µm)	MOV (V)	Translation Factor T (nm/°)				
		Comb-Drive 1	Comb-Drive 2	Comb-Drive 3	Comb-Drive 4	Comb-Drive 5
0.5	1.8	1.9	3.6	4.4	4.5	3.5
0.6	2.2	2.8	4.6	5.4	4.9	3.7
0.7	2.7	3.7	5.7	6.2	5.8	2.9
0.8	3.2	4.2	4.2	6.6	5.9	3.7
1	4.4	5.9	8.1	8.2	6.9	4.1

## Data Availability

Data are contained within the article.
